# Trade-off between Positive and Negative Design of Protein Stability: From Lattice Models to Real Proteins

**DOI:** 10.1371/journal.pcbi.1000592

**Published:** 2009-12-11

**Authors:** Orly Noivirt-Brik, Amnon Horovitz, Ron Unger

**Affiliations:** 1 Department of Structural Biology, Weizmann Institute of Science, Rehovot, Israel; 2 The Mina and Everard Goodman Faculty of Life Sciences, Bar-Ilan University, Ramat-Gan, Israel; University of California San Diego, United States of America

## Abstract

Two different strategies for stabilizing proteins are (i) positive design in which the native state is stabilized and (ii) negative design in which competing non-native conformations are destabilized. Here, the circumstances under which one strategy might be favored over the other are explored in the case of lattice models of proteins and then generalized and discussed with regard to real proteins. The balance between positive and negative design of proteins is found to be determined by their average “contact-frequency”, a property that corresponds to the fraction of states in the conformational ensemble of the sequence in which a pair of residues is in contact. Lattice model proteins with a high average contact-frequency are found to use negative design more than model proteins with a low average contact-frequency. A mathematical derivation of this result indicates that it is general and likely to hold also for real proteins. Comparison of the results of correlated mutation analysis for real proteins with typical contact-frequencies to those of proteins likely to have high contact-frequencies (such as disordered proteins and proteins that are dependent on chaperonins for their folding) indicates that the latter tend to have stronger interactions between residues that are not in contact in their native conformation. Hence, our work indicates that negative design is employed when insufficient stabilization is achieved via positive design owing to high contact-frequencies.

## Author Summary

Most proteins are functional only in their native states. The stability of the native state of proteins is, therefore, of paramount importance both *in vivo* and for many biotechnological applications *in vitro*. Protein stability is determined by the difference between the free energies of the native and non-native states. It follows that protein stabilization can be achieved via two different strategies: (i) ‘positive design’ by introducing favorable interactions between residues in contact in the native state; and (ii) ‘negative design’ by introducing unfavorable interactions between residues in contact in the non-native states. Here, we ask when is one strategy favored over the other. We show that ‘positive design’ is favored when interactions that stabilize the native state are rarely found in the non-native states whereas ‘negative design’ is favored when the interactions that stabilize the native state are also common in the non-native states. We also show that correlated mutations, i.e. mutations at one site that compensate for effects of mutations at other sites, tend to be associated with ‘negative design’. Analysis of protein sequence data shows that a higher incidence of correlated mutations is found in protein families with native states that are not stable or difficult to reach.

## Introduction

Protein stabilization can be achieved via two different strategies: (i) ‘positive design’ in which the native state is stabilized; and (ii) ‘negative design’ in which non-native states are destabilized [1]–[3]. Positive design can be achieved by introducing favorable pairwise interactions between residues that are in contact in the native state whereas negative design can be achieved by introducing unfavorable pairwise interactions between residues that are in contact in non-native conformations of the protein. The factors that favor employing one strategy over the other (or some combination of both strategies) are not known. For example, it is possible that certain features of a protein's native structure such as its secondary structure content or contact-order [4] bias the choice of which particular strategy is employed. Here, we explore this question with respect to lattice models of proteins and then show that the principles that we have discovered also apply to real proteins. Although lattice models of proteins ignore many important details, they have been used successfully for elucidating general principles of protein folding and stability [5]–[8] and addressing evolutionary questions [9]–[11]. In particular, such models have the advantage that in certain cases all the possible conformations in the ensemble can be enumerated and, therefore, preferential design strategies for certain protein conformations may be identified.

The stability, dynamics and function of proteins are determined by both short- and long-range pairwise interactions. Long-range interactions are manifested, for example, in the energetic coupling between distant ligand-binding sites in allosteric proteins owing to conformational changes that are propagated from one site to another. The strength of both direct (short-range) and indirect (long-range) pairwise interactions can be analysed experimentally using the double-mutant cycle (DMC) method [12]. Recently, we introduced a computational version of DMCs for analysis of pairwise interactions in lattice models of proteins [13]. Computational DMC analysis can be easily employed in an exhaustive manner to determine the strength of interaction between all possible residue pairs in a lattice model in contrast with experimental DMC analysis that must be restricted to a relatively small number of residue pairs owing to the prohibitive amount of work involved. Using this computational DMC approach, we previously discovered that the strength of both short- (i.e. between residues in contact in the native state) and long-range (i.e. between residues not in contact in the native state) pairwise interactions changes in a linear fashion with increasing ‘contact-frequency’, a term defined for each pair of residues in a sequence that corresponds to the fraction of states in the conformational ensemble of the sequence in which that pair of residues are in contact [13],[14]. In other words, a pair of residues that are in contact in many conformations available to the chain has a high contact-frequency whereas a pair of residues that are rarely in contact has a low contact-frequency. A protein fold can, therefore, be characterized by the average contact frequency of all the residue pairs in contact in that fold. Here, we show for lattice models that positive design is favored when this average ‘contact frequency’ is low whereas negative design is favored when this average ‘contact frequency’ is high. A mathematical derivation of this result indicates that it is general and, thus, likely to hold also for real proteins.

Negative design in the lattice models is also found to be associated with a higher incidence, on average, of correlated mutations, i.e. mutations at one site that tend to be accompanied by other mutations at a second site. Correlated mutations are assumed to be due to selective pressure to maintain protein structure or function and have, therefore, been used for prediction of 3D protein structure [15],[16], allosteric pathways [17],[18] and protein-protein interactions [19],[20] and in protein design [21]. Correlated mutations in real proteins, however, also reflect common ancestry [22]–[24] whereas in our lattice models this concern is obviated. Here, by applying correlated mutation analysis we show that proteins likely to have a higher average ‘contact-frequency’, such as disordered proteins, also have a higher incidence of correlated mutations. These results strengthen our conclusion that ‘contact-frequency’ is an important factor in determining the design strategy of real proteins.

This paper is organized as follows. First, we show that the effects on stability of positive and negative design in lattice models are both linearly dependent, but with opposite sign, on the contact-frequency and that there is a strong trade-off between them. We then provide a general (not lattice specific) mathematical derivation supporting these claims. An analysis of correlated mutations in sequences selected for stability of a lattice fold that follows next shows that the density of correlated mutations increases with increasing contact-frequency. Finally, we show that a similar trend is likely to exist in real proteins by analyzing correlated mutations in proteins that fold with difficulty and are suspected to have higher contact-frequencies.

## Results

### Selection in lattice models

**Figure 1:**
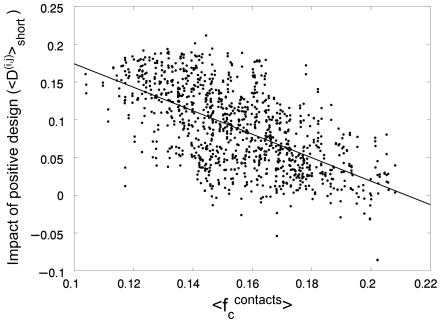
Relationship between the impact of positive design on the stability of different lattice folds and their respective average contact-frequencies. The values of a measure of the effect of positive design of stability, <D^(i,j)^>_short_, for the 1081 different folds of 25 residue-long sequences on a 5×5 lattice are plotted against their respective average contact-frequencies, 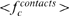. A linear correlation is observed with *r* = −0.6082 and a *P*-value<0.0001.

Sets of 25 residue-long sequences that share a particular native state were generated with and without selection for native state stability. The native states of the sets that were formed (termed SBSS) correspond to each of the 1081 compact folds on a 5×5 lattice. The average perturbation energy (ΔΔG_per_) was then calculated for each pair of positions i and j in an alignment and the difference, D^(i,j)^, in the average perturbation energies for that pair of positions in the alignments with and without selection was determined. The average value of D^(i,j)^ was then calculated for all pairs of positions in contact in a particular native conformation, <D^(i,j)^>_short_, and for all pairs that form long-range interactions in that conformation, <D^(i,j)^>_long_. Two positions are defined as forming a long-range interaction in a particular conformation if there is no path formed by residues in contact in that conformation that connects them (Figure S1). For example, if residue A is in contact with residue B and residue B is in contact with residue C then we do not consider residues A and C to be involved in a long-range interaction. Hence, the number of long-range interactions varies slightly between folds since the paths that connect residues in contact depend on the specific conformation. In the case of a compact conformation on a 5×5 lattice, the number of long-range interactions is 108
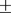
3 whereas the number of pairs in contact is always 16. It is important to note that all pairs of positions with a contact-frequency of zero (in the case of a square lattice, for example, residues at positions with the same parity cannot be in contact) are not considered in this analysis since their ΔΔG_per_ equals zero by definition. The values of <D^(i,j)^>_short_ of different folds were found to be correlated with their respective average contact-frequencies (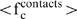). It can be seen in Figure 1 that the value of <D^(i,j)^>_short_ decreases when the corresponding value of 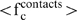 for that fold increases (r = −0.608; *P*-value<0.0001). Smaller values of <D^(i,j)^>_short_ reflect a smaller contribution of pairs in contact to the gain in stability upon selection. Surprisingly, we discovered that some native states have zero or even negative <D^(i,j)^>_short_ values. Such values are found when the value of 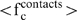 is large. This observation indicates that positive design is almost a negligible factor when stabilizing native states with a very high average contact-frequency.

**Figure 2 pcbi-1000592-g002:**
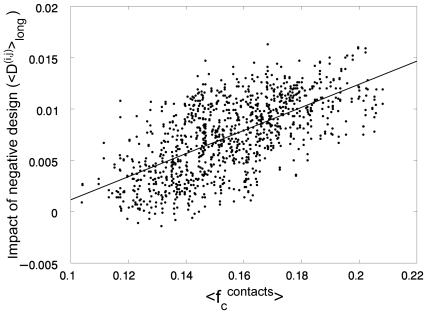
Relationship between the impact of negative design on the stability of different lattice folds and their respective average contact-frequencies. The values of a measure of the effect of negative design of stability, <D^(i,j)^>_long_, for the 1081 different folds of 25 residue-long sequences on a 5×5 lattice are plotted against their respective average contact-frequencies, 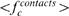. A linear correlation is observed with *r* = 0.6390 and a *P*-value<0.0001.

**Figure 3:**
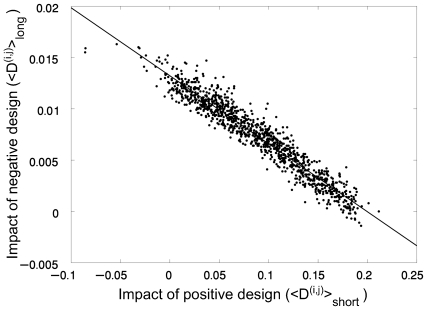
Trade-off between the effects of positive and negative design on the stabilities of different lattice folds. The values of a measure of the effect of negative design of stability, <D^(i,j)^>_long_, for the 1081 different folds of 25 residue-long sequences on a 5×5 lattice are plotted against their respective values of a measure of the effect of positive design of stability, <D^(i,j)^>_short_. A linear correlation is observed with *r* = −0.96 and a *P*-value<0.0001.

We also examined whether a correlation exists between the contribution of negative design to stability and the average contact-frequency. The correlation between <D^(i,j)^>_long_ and 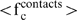 is shown in Figure 2 and found to be significant (r = 0.639; *P*-value<0.0001). It may also be seen in Figure 2 that negative design is hardly used for stabilizing native states with a very low average contact-frequency. In Figure 3, <D^(i,j)^>_short_ for each fold is plotted against the corresponding value of <D^(i,j)^>_long_. The correlation observed is almost perfect (r = −0.96, *P*-value<0.0001), thereby revealing the strong trade-off between the two strategies of positive and negative design.

### General derivation of the relationships between the contributions of positive and negative design of protein stability and contact-frequency

The results shown in Figures 1–3 for a specific lattice model prompted us to examine whether a general derivation can be obtained for the linear dependence of the contributions of positive and negative design to protein stability on contact-frequency. The starting point for the following such derivation is the previously derived [13] linear relationship between the perturbation energy and Boltzmann-weighted contact-frequency:
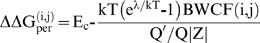
(1)where E_c_ is the energy of a contact that was removed (see Table 1 in [13]), 
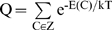
 (Z is the ensemble of all possible conformations and E(C) is the energy of a conformation), 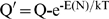 (E(N) is the energy of the native state), λ is the contact energy of the residue types found at positions i and j (see Table 1 in [13]), *T* is the temperature and *k* is the Boltzmann constant. The Boltzmann-weighted contact-frequency, BWCF(i, j), is calculated by multiplying each occurrence of a contact by the Boltzmann weight of the conformation in which it occurs. Eq. (1) can be written in a simplified and approximate form, as follows:
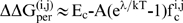
(2)where 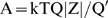 and 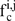 is the contact-frequency that is not Boltzmann-weighted. Given an alignment of sequences with the same native fold, one may express the average perturbation energy for a pair of positions i and j in the alignment, 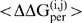, as follows:
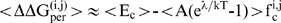
(3)The difference, D^(i,j)^, in the average perturbation energies with and without selection was determined for every relevant pair of positions in the alignments. Inspection of Eq. (3) shows that D^(i,j)^ for positions i and j in the alignment is equal to:
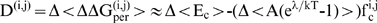
(4)where Δ designates the differences in these terms with and without selection. The average of D^(i,j)^ over all the pairs of positions i and j that form direct short-range native-state contacts, <D^(i,j)^>_short_, can therefore be written using Eq. (3), as follows:
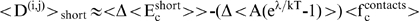
(5)where 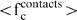 is the average contact-frequency of the short-range native-state contacts, 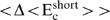 is the average of Δ<E_c_> over all the pairs of positions i and j that form direct short-range native-state contacts and 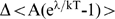 is assumed to be the same for all these pairs. Eq. (5) describes a linear relationship with a negative slope between <D^(i,j)^>_short_, which is a measure of the impact of positive design on stability, and 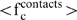 as observed in Figure 1 for the lattice model. An expression similar to Eq. (5) for the case of long-range interactions can be written, as follows:
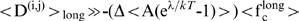
(6)Eq. 6 is similar in form to Eq. (5) except that E_c_ = 0 as it is for the case of long-range interactions. Given that the sum of all the contact-frequencies is equal to some constant, α, we can write:
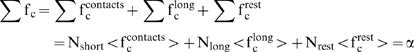
(7)where 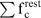
corresponds to the sum of contact-frequencies of all the residue pairs that do not form short- or long-range interactions as defined above. Eqs. (6) and (7) can be combined to yield:
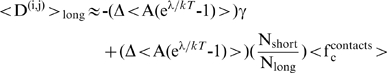
(8)where 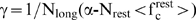. Eq. (8) describes a linear relationship with a positive slope between <D^(i,j)^>_long_, which is a measure of the impact of negative design on stability, and 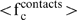 as shown in Figure 2. Given the simplifying assumptions we made that 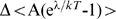 is the same for all the relevant residue pairs and that the contact-frequency is not Boltzmann-weighted, it is not surprising that the correlations shown in Figures 1 and 2 for a specific model are noisy. The above derivations do show, however, that these correlations are general and not specific for particular lattice models and, thus, likely to hold for real proteins.

**Table 1:** Comparison between the correlated mutation densities averaged for all the alignments corresponding to the different sets examined.

Data set^a^	Number of alignments	Average density of correlated mutations	S.D.
‘Control’ proteins	432	0.0018	0.007
GroEL-dependent substrates (class I)	35	0.0027	0.007
GroEL-dependent substrates (class II)	110	0.0040	0.006
GroEL-dependent substrates (class III)	77	0.0051	0.009
Intrinsically unstructured proteins	72	0.0103	0.023

a Each data set is comprised of sequence alignments generated using a reference sequence belonging to one of the five groups listed.

### Analysis of correlated mutations in lattice models

**Figure 4:**
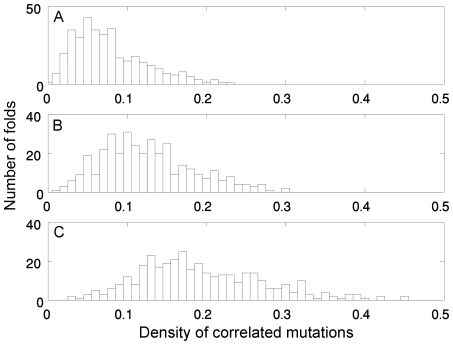
Distributions of densities of correlated mutations at positions involved in long-range interactions for different classes of lattice folds with increasing values of average contact-frequency. The 1081 different folds of 25 residue-long sequences on a 5×5 lattice were ordered according to their average contact frequency, (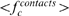), and then divided into three equal-sized groups comprising the folds with the lowest (A), in between (B) and highest (C) values of 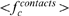, respectively. It can be seen that the density of correlated mutations tends to increase as the average contact-frequency of the fold increases.

The different SBSS corresponding to the 1081 different 5×5 lattice folds were subjected to correlated mutation analysis in order to determine whether there is a connection between this phenomenon and the stabilization strategy. The correlated mutation analysis was able to identify all the 16 pairs of positions that are in contact in all the 1081 different folds except for some rare cases in which one or two contacts were not detected. In the case of the long-range interactions, the strength of the correlated mutations signal for a given fold was found to depend on the average contact frequency of its contacts. The different folds were divided into three equal-sized classes corresponding to different ranges of values of 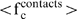 and the distributions of densities of correlated mutations (see Methods) at positions involved in long-range interactions were plotted for each class (Figure 4). Although the distributions are overlapping, a clear trend is observed that the average density of long-range correlated mutations increases with increasing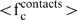. The correlation coefficient between the density of correlated mutations at positions involved in long-range interactions and 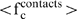 is 0.626 with a *P*-value<0.00001 (not shown). Hence, correlated mutations at positions involved in long-range interactions appear to be associated with negative design that is also found when 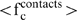 is high.

### Analysis of correlated mutations in real proteins

The apparent connection between employing negative design and prevalence of correlated mutations at positions involved in long-range interactions enables us to expand our analysis to real protein data. Given that the calculation of the contact-frequency parameter for a large number of real proteins is impractical owing to the huge size of their conformational spaces, we decided to look into groups of proteins for which there is good reason to assume that their average contact-frequency is high. We analysed two sets of proteins that are likely to have a high average contact-frequency of their contacts. The first set contains intrinsically unstructured proteins (IUP) that populate many conformations and are, therefore, likely to have relatively high values of contact-frequency since individual contacts probably stabilize many different conformations. The second set is based on the GroEL-interacting proteins found by Hartl and co-workers [25]. These proteins were divided into classes I to III with increased dependency on the GroE chaperonin system for folding correctly [25]. A possible reason that these proteins have low folding propensities is their relatively high contact-frequency. In addition, we generated a third set of proteins as a control (see Methods) for the other sets. Given that structural information is not available for disordered regions of proteins and, also, for many of the other sequences, we could not distinguish here between correlated mutations at positions involved in short-range contacts and those involved in long-range interactions. However, this does not affect our conclusions as the fraction of correlated mutations at positions in contact is lower than 20% [24] and is likely to be approximately equal in all the sets. Hence, the variation in the densities stems mostly from the long-range correlations.

**Figure 5:**
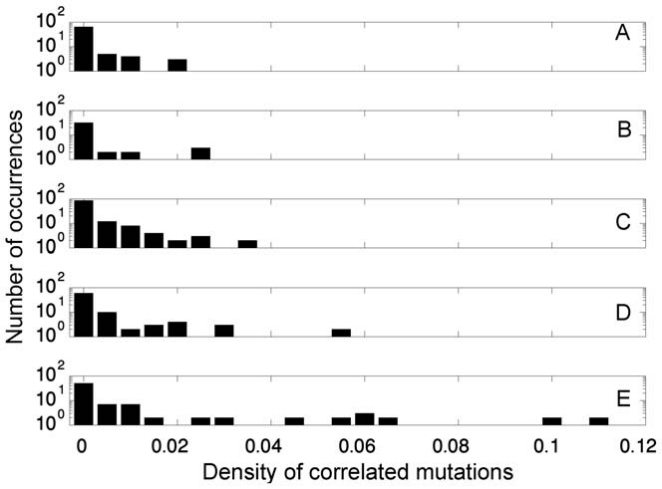
Distributions of correlated mutation densities in the case of the five different sets of real proteins examined in this study. The densities of correlated mutations were calculated for the sets of control proteins (A), classes I (B), II (C) and III (D) of the GroEL-interacting proteins and the intrinsically unstructured proteins (E). It can be seen that the density of correlated mutations of these sets increases with the increasing likelihood that their average ‘contact-frequency’ has increased.

The distributions of correlated mutation densities calculated using the tree-based method [24] are shown in Figure 5 for the sequence alignments based on the set of disordered proteins, the three classes of GroEL-dependent proteins and the set of other randomly chosen control proteins (equal in number to that of the IUP-based set). The averages and standard deviations of all the sets are given in Table 1. It can be seen that the average density of correlated mutations is lowest in the case of the control set of proteins, it is higher in the case of the three classes of GroEL-dependent substrates (and increases from class I to III) and is highest for the IUP-based set. This trend is observed only when comparing the average correlated mutation densities of the sets but it is important to note that correlated mutation analysis of real proteins is much noisier than that of lattice model proteins due to the larger alphabet size, errors in sequence alignment and evolutionary background and, therefore, these observations are significant. It should also be noted that fewer correlations were obtained in the case of real proteins as compared with lattice models as the former were detected using the tree-based method that was developed to filter out evolutionary noise and is more stringent.

## Discussion

A key observation in this study (Figure 3) is that the balance between the contributions of positive and negative design to the stability of different lattice folds varies despite the fact that all the sequences were subjected to the same selection pressure. It is important to note that mutations that affect short-range interactions tend to have much larger effects on stability than those that affect long-range interactions [13], suggesting that positive design should be much more common than negative design. However, we find that some folds underwent stabilization by using only negative design. This unexpected result indicates that positive design has limited ability to stabilize certain folds and that negative design compensates for that in cases of such folds (Figure 3). Our results show that folds that can be stabilized by both positive and negative design are distinguished from those that are stabilized mostly by negative design in their average contact-frequency. Folds with low contact-frequency can be stabilized by both positive and negative design whereas those with high contact-frequency can be stabilized mostly by negative design. These results suggest that contact-frequency determines the stabilization potential of different folds and that certain folds are, therefore, more likely to emerge under difficult folding conditions such as extreme temperatures.

The analysis in this paper is based on the premise that stabilization of short-range contacts reflects positive design whereas stabilization of long-range interactions reflects negative design. In lattice models, this assumption is correct since the energy of any native state is determined only by its contacts and, therefore, any stabilization due to long-range interactions must stem from destabilization of non-native states (i.e. negative design). In the case of real proteins, however, this assumption is not necessarily correct since long-range (e.g. electrostatic) interactions can also stabilize the native state. However, the correlated mutation results that we obtained for both the lattice models and real proteins showed the same trend and, therefore, we assume that the correlated mutations that are mostly between distant positions reflect negative design.

It is interesting that two different mechanisms for thermostabilization have also been revealed by comparing mesophilic proteins with their thermophilic homologs [26]. One mechanism termed “structure-based” is reflected in structure compactness and appears in proteins that originated in extreme environments. The second mechanism termed “sequence-based” is reflected in a bias of the amino acid composition toward more charged residues and is found in proteins that originated as mesophiles but later had to adapt to higher temperatures. Hence, both the findings here and the work of Berezovsky *et al.* [26] indicate that certain structural (e.g. topological) features of proteins dictate their stabilization potential and that tinkering with sequence can compensate for the lack of such structural features. Thermostability has been attributed previously to amino acid composition [27]–[29] but by having all the lattice model sequences in our work share the same composition we were able to identify a purely structural basis for stabilization.

In conclusion, in this study we subjected lattice model proteins to selection for stability and showed that the balance between positive and negative design strategies differs for each fold and depends on the average ‘contact-frequency’ of that fold. The use of negative design is found to increase with increasing values of the average ‘contact-frequency’ of the respective fold. Our results, therefore, indicate that each fold has its own stabilization potential that limits its ability to adapt to extreme conditions. We also showed that negative design in lattice models can be identified by correlated mutation analysis and is reflected in higher values of correlated mutation densities. This trend was also found in correlated mutation analysis of real proteins when comparing intrinsically unfolded proteins and chaperonin-dependent protein substrates to other control proteins. Thus, we conclude that stabilization of real proteins with high values of average contact-frequency tends to rely more on negative design and is reflected in higher densities of correlated mutations.

## Methods

### 

#### The lattice model of proteins

A 2D lattice model similar to the one described before [13] was used. Here, we describe the main features of the model relevant to the current work. The protein sequence consists of an alphabet of four amino acids: hydrophobic (H), neutral polar (P), positively charged (+) and negatively charged (−). All the sequences that were generated have 25 residues and the same composition of 40% H, 28% P, 16% (+) and 16% (−) that corresponds roughly to that of soluble proteins in the PDB. The energy of a sequence in a specific lattice conformation, E(C), was calculated by summing all the pairwise contact energies, e_ij_, between neighboring lattice points (excluding consecutive residues in the sequence which are always neighbors), as follows:
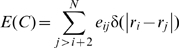
(9)where 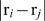 is the distance in lattice units between residues i and j that are separated in sequence by at least two residues and 
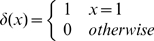
. The pairwise interaction energies (e_ij_) are as before [13] and reflect in a qualitative manner the strengths of interactions between different types of amino acids.

The free energy of folding, ΔG, of the native conformation of a sequence was calculated using [5]:
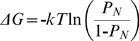
(10)where P_N_ is the probability that the chain is in its native state N. This probability is given by: 
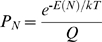
, where 
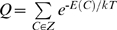
 (Z is the ensemble of all possible conformations on the relevant lattice), E(N) is the energy of the native conformation, T is the temperature and *k* is the Boltzmann constant. A value of 1 was used for *k*T. The energies of all possible 1081 non-symmetric conformations of a given 25 residue-long sequence that fit a 5×5 lattice were calculated and the conformation with the lowest energy, if a single such one exists, was considered as its native conformation.

#### Generation of structure-based sequence sets (SBSS)

Sets of sequences that have the same native conformation were generated. Two kinds of sets were generated for each of the 1081 lattice conformations. In the first set, the only requirement was that all the sequences comprising the set have the same particular native conformation. In the second set, we required that the free energy of folding to the native state of the selected sequences is lower than some threshold value. Sets of the first type can be generated easily by classifying random sequences to different SBSS according to their native conformation. Sets of the second type could not be generated rapidly using this simple procedure and, therefore, we used a Monte Carlo (MC) maximization process of the following function:
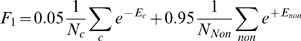
(11)where N_c_ and N_non_ are the total number of contacts and non-contacts in the specific conformation, respectively. In each step of the MC process, two residues in the sequence were randomly swapped and the swap was accepted if the Metropolis criterion [30] was met. The MC process was stopped when a sequence with the requested native state and with ΔG<ΔG_threshold_ was found. We will refer to the SBSS that were generated by procedures (i) and (ii) as the sets without and with selection, respectively. Each set contained between 50–64 sequences. The average ΔG of folding for all the sequences in the sets without selection is approximately 2.6±0.6 and, therefore, the threshold, ΔG_threshold_, for selecting sequences with stable native folds was set to zero.

#### Calculation of perturbation energies

We calculated a perturbation energy, ΔΔG_per_ = ΔG_wt_−ΔG_m_, for every possible pair of positions in each sequence where ΔG_wt_ and ΔG_m_ are the respective free energies of folding of the wild-type sequence before and after a particular short- or long-range pairwise interaction is ‘turned off’ but without affecting any other interaction. Under ideal circumstances [31], the pairwise interaction energy (or coupling energy) determined experimentally using DMC provides a good estimate of the perturbation energy that can only be determined by computation. In the case of lattice models, the values of the computationally derived pairwise coupling energies from DMC are essentially identical to those of the corresponding perturbation energies [13], thus justifying the use of perturbation energies to estimate the strength of pairwise interactions. The ΔΔG_per_ for each pair of positions i and j was determined for each sequence in a SBSS and the average value, 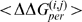, for that pair of positions in all the sequences in the SBSS was calculated. The difference between 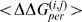 for the sets with and without selection was defined as:
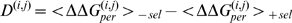
(12)


#### Calculation of the average ‘contact-frequency’ of a lattice native state

The fraction of conformations in the ensemble in which residues at two positions in a sequence are in contact is termed the ‘contact frequency’. The contact frequency is sequence-independent and is a function only of the length of the protein, the positions of the two residues in the sequence and the lattice dimensions [14]. It is also possible, however, to define a Boltzmann-weighted contact frequency [13] that is sequence-dependent as each occurrence of a contact is multiplied by the Boltzmann weight of the conformation in which it occurs. The average ‘contact frequency’, 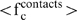, which is calculated for all the pairs of positions that are in contact in a specific native state is not Bolzmann-weighted and is, therefore, sequence-independent but can be viewed as a property of all the sequences that adopt that particular native state.

#### Generation of real proteins data sets

Three data sets of real protein sequence alignments were generated: (i) the IUP set; (ii) the GroEL-interacting proteins set; and (iii) a control set of alignments of proteins that does not include any members of the first two sets and their homologs. The IUP data set was generated by downloading the DisProt database version 4.8 (http://www.disprot.org/) [32] and selecting all sequences that contain disordered regions of at least 50 residues long that have a UniProt [33] accession number. These sequences were used as references for generating the IUP-based alignments. The GroEL-interacting proteins data set was generated by downloading from the PEDANT database (http://pedant.gsf.de/links.jsp) the sequences of GroEL-interacting proteins reported by Kerner *et al.* [25]. This data set contains 252 proteins that are divided into classes I to III (with increasing dependency on GroEL for folding) that comprise 38, 126 and 84 proteins, respectively, and four other proteins whose class was not determined. The protein P00810 (which belongs to class I) was excluded from our analysis as it is a β-lactamase that is not part of the *Escherichia coli* (*E. coli*) K12 proteome. These sequences were used for generating the alignments based on GroEL-interacting proteins. The control set was generated by downloading the sequences of the full proteome of the K12 strain of *E. coli* (corresponding to accession number NC_000913.1) from the Refseq database ftp://ftp.ncbi.nih.gov/genomes/Bacteria/Escherichia_coli_K_12_substr__MG1655/ [34]. Homologs of members of the IUP or GroEL-interacting proteins sets with an E-value smaller than 0.1 were identified using BLAST [35] and eliminated from this control set. The remaining sequences were used as references for generating the control alignments.

Multiple sequence alignments (MSA) corresponding to the above three sets were generated by searching the UniProt database [33] using BLAST [35] for up to 250 homologs (with an E-value smaller than 1) of each reference sequence. Paralogs were filtered out and MSAs were generated using CLUSTAL W version 2.0.10 [36] as described [24]. It is important to note that alignments containing less than 50 sequences or with an average pairwise sequence identity below 45% were discarded. A total of 72 IUP-based alignments, 222 GroEL-interacting proteins-based alignments (divided into 35, 110 and 77 alignments corresponding to the three different classes mentioned above) and more than 400 control alignments (based on references randomly selected from the 3339 *E. coli* control proteins) were generated.

#### Correlated mutation analysis

Correlated mutation analysis was carried out for both real protein sequences and lattice model sequences. In the case of the real proteins, our tree-based method [24] was applied in order to reduce the extent of evolutionary noise and all pairs of positions with a *P*-value equal to or smaller than 0.05 were considered as coupled. In the case of the lattice sequences, a random shuffling procedure was used as the sequences were selected randomly and do not share a common ancestor and, therefore, there was no need for filtering evolutionary noise using our tree-based method. The correlated mutation density is defined as the number of pairs of positions that were found to have a significant correlation divided by the total number of possible pairs of positions. Note that conserved positions or positions at which more than 10% of the sequences in the alignment have a gap (this is relevant only to real proteins) were not considered in the analysis. In addition, the correlated mutation densities of the intrinsically unstructured proteins (IUP)-based alignments were calculated using only the disordered positions.

## Supporting Information

Figure S1 Scheme of a lattice model showing examples for (i) short-range interactions between residues in contact and (ii) long-range interactions between residues that are not in contact either directly or indirectly. Examples for pairs of residues involved in short-range interactions (e.g. 17 and 24) are indicated by the red line that connects the two residues in contact. Residues 8 and 24, for example, are in indirect contact since there is a path formed by residues in contact that connects them (8-19-14-17-24). By contrast, residues 2 and 13, for example, that are connected by the dashed arrow are defined as being involved in a long-range interaction since there is no path formed by residues in contact that connects them.
(0.61 MB TIF)
