# Supplementary figures and images for: Trade-off between Positive and Negative Design of Protein Stability: From Lattice Models to Real Proteins
Source: PLoS Comput Biol. 2009 Dec 11;5(12):e1000592. doi: 10.1371/journal.pcbi.1000592 (PMC2781108; doi:10.1371/journal.pcbi.1000592)

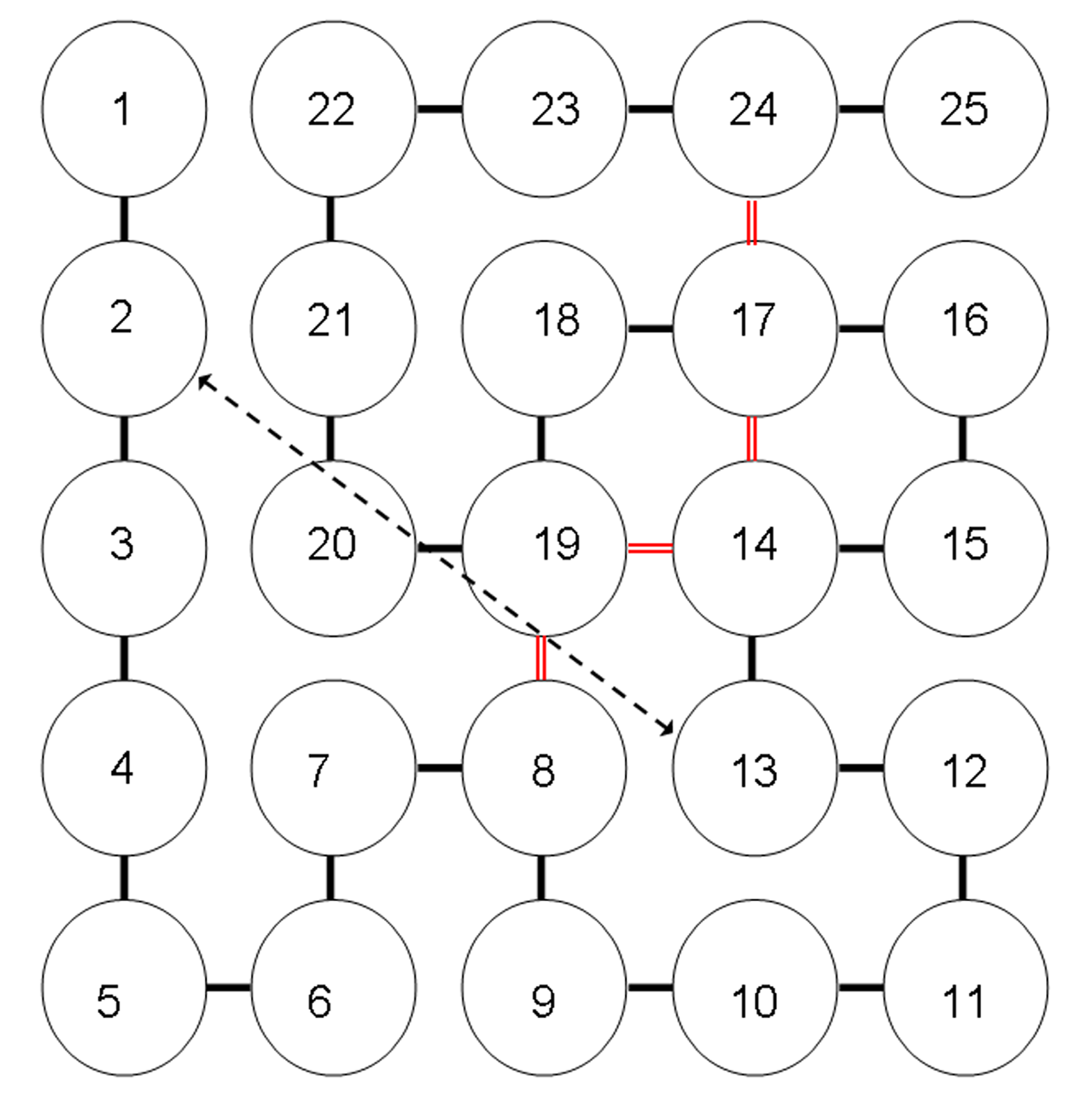

Supplement: Figure S1 — Scheme of a lattice model showing examples for (i) short-range interactions between residues in contact and (ii) long-range interactions between residues that are not in contact either directly or indirectly. Examples for pairs of residues involved in short-range interactions (e.g. 17 and 24) are indicated by the red line that connects the two residues in contact. Residues 8 and 24, for example, are in indirect contact since there is a path formed by residues in contact that connects them (8-19-14-17-24). By contrast, residues 2 and 13, for example, that are connected by the dashed arrow are defined as being involved in a long-range interaction since there is no path formed by residues in contact that connects them. (0.61 MB TIF) [file pcbi.1000592.s001.tif]
